# A Computational Neural Model for Mapping Degenerate Neural Architectures

**DOI:** 10.1007/s12021-022-09580-9

**Published:** 2022-03-29

**Authors:** Zulqarnain Khan, Yiyu Wang, Eli Sennesh, Jennifer Dy, Sarah Ostadabbas, Jan-Willem van de Meent, J. Benjamin Hutchinson, Ajay B. Satpute

**Affiliations:** 1 Department of Electrical & Computer Engineering, College of Engineering, Northeastern University, Boston, 02115 MA USA; 2 Department of Psychology, College of Science, Northeastern University, Boston, 02115 MA USA; 3 Khoury College of Computer Sciences, Northeastern University, Boston, 02115 MA USA; 4 Department of Psychology, University of Oregon, Eugene, 97403 OR USA

## Abstract

Degeneracy in biological systems refers to a many-to-one mapping between physical structures and their functional (including psychological) outcomes. Despite the ubiquity of the phenomenon, traditional analytical tools for modeling degeneracy in neuroscience are extremely limited. In this study, we generated synthetic datasets to describe three situations of degeneracy in fMRI data to demonstrate the limitations of the current univariate approach. We describe a novel computational approach for the analysis referred to as neural topographic factor analysis (NTFA). NTFA is designed to capture variations in neural activity across task conditions and participants. The advantage of this discovery-oriented approach is to reveal whether and how experimental trials and participants cluster into task conditions and participant groups. We applied NTFA on simulated data, revealing the appropriate degeneracy assumption in all three situations and demonstrating NTFA’s utility in uncovering degeneracy. Lastly, we discussed the importance of testing degeneracy in fMRI data and the implications of applying NTFA to do so.

**Electronic Supplementary Material:**

The online version of this article (10.1007/s12021-022-09580-9) contains supplementary material, which is available to authorized users.

## Introduction

Degeneracy refers to the capability of different structures to produce the same effects (Edelman & Gally, 2001; Tononi et al., 1999; Whitacre, 2010). For example, different sets of codons in genetics can produce the same phenotype (Konopka, 1985). Different ion channels - more than are strictly necessary - are used to tune the firing rate of neurons (Drion et al., 2015). Different distributions of neural modulators and circuit parameters nonetheless produce the same rhythmic activity in a neural circuit (Gutierrez et al., 2013; Gutierrez & Marder, 2014). Simple motor behaviors, like finger tapping, may also be produced by an abundance of distinct motor pathways (Bernstein, 1966; Wolpert, 2003; Seifert et al., 2016; Latash, 2012). In functional neuroanatomy, degeneracy refers to the notion that the brain may have multiple solutions or a surplus of neural pathways to produce the same mental state or behavior (Price & Friston, 2002; Friston & Price, 2003; Sajid et al., 2020). Indeed, computational simulations show that degeneracy is high in networks with high complexity such as the brain (Tononi et al., 1994; Tononi et al., 1996), in which multiple distinct, parallel structural pathways may lead from a source node to a destination node. Such an architecture enables a degree of robustness to changes in the neural environment (e.g. due to tissue damage) (Price & Friston, 2002; Sajid et al., 2020). The concept of degeneracy may overlap with redundancy because they both suggest there are multiple solutions that can produce the same output, however they differ in the flexibility for the system to choose which solution to produce the outcome (Edelman & Gally, 2001; Friston & Price, 2003; Marder & Taylor, 2011; Sajid et al., 2020).

In cognitive neuroscience, degeneracy suggests there might be systematic sources of variance across trials or individuals that are of interest for the brain-behavior relationship. For example, two individuals may use different neural pathways to perform the same task, or one individual may use different neural pathways in different moments when performing a task. Commonly used analytical approaches often treat such variation across trials within a condition and across individuals within a sampled group of participants as error. For example, functional neuroimaging studies that examine task-dependent changes in functional activation often estimate parameters assuming invariance across trials or participants. Offering a bit more flexibility, recent machine learning approaches have also been applied to functional neuroimaging data (e.g. multivoxel pattern analysis)(Kriegeskorte et al., 2006; Haxby, 2012), however, these approaches commonly rely on supervised analytical approaches that imply a common neural activation pattern for trials in the same task (Azari et al., 2020). In both cases, summaries are calculated either across participants, trials, or both in order to increase signal-to-noise ratios, and residual variance is assumed to provide an estimate of error for calculating inferential statistics. However, in doing so, these approaches are assuming a non-degenerate functional architecture *a priori*. As a result, little is known about the extent to which these assumptions prevail vs. the extent to which there is degeneracy in functional neuroanatomy.

Uncovering degeneracy requires analytical tools that are explicitly designed for this purpose. If the brain provides multiple solutions to complete a given task, then functional activation patterns in a given study may depend on the participant and moment in time (i.e. by stimulus or trial) in ways that are unbeknownst to investigators. Thus, it is important to develop an analytical approach that can identify sources of structure in signal with minimal supervision - that is, without relying on strong *a priori* assumptions of investigators of how functional activity ought to relate with task performance. Here, we propose a novel computational model, referred to as Neural Topographic Factor Analysis (NTFA), to examine degeneracy in functional neuroanatomy. Our model is built off of earlier topographic factor analysis approaches (Manning et al., 2014b) and takes as input individuated segments of 4D fMRI timeseries data with labels for participant and trial. It does not require knowledge about the attributes of participants (demographic, personality, genetic, etc.), nor does it require knowledge about how trials sort into conditions. NTFA learns a low-dimensional representation - or an embedding - of functional activity for each participant and trial on the basis of shared patterns of neural activation from segments of data. These embeddings provide a simple, readily visualizable depiction of whether and how neural responses during a task vary across participants, trials, and participant by trial combinations.

In this paper, our goal is to validate NTFA using a simulation approach. Computational simulations are critical to test whether novel computational models are capable of performing as expected in principle, that is, under conditions with a known ground truth. In practice, the data generating mechanisms for functional neuroanatomy are rarely, if ever, known. That is why it is of particular importance in cognitive neuroscience to develop modeling approaches that are capable of providing insight as to whether there is likely to be degeneracy in functional neuroanatomy from the data alone and with minimal supervision. Using computational simulations, we first demonstrate the considerable shortcomings of applying the most commonly used “univariate” activation-based analytical approach in fMRI data analysis when there is degeneracy. In the typical form of this analysis, a general linear model is used to determine whether functional activity in a given voxel or brain region (i.e. set of voxels) is greater during trials from one experimental condition relative to a baseline condition. We then implement NTFA on simulated datasets with minimal assumptions about whether trials ought to be nested into particular task conditions, or participants into particular groups. Our deliverable is a demonstration of the ability of NTFA to recover embeddings that reveal degeneracy, and non-degeneracy, in simulated 4D timeseries data with topological structure (e.g. as in fMRI data).

**Fig. 1:**
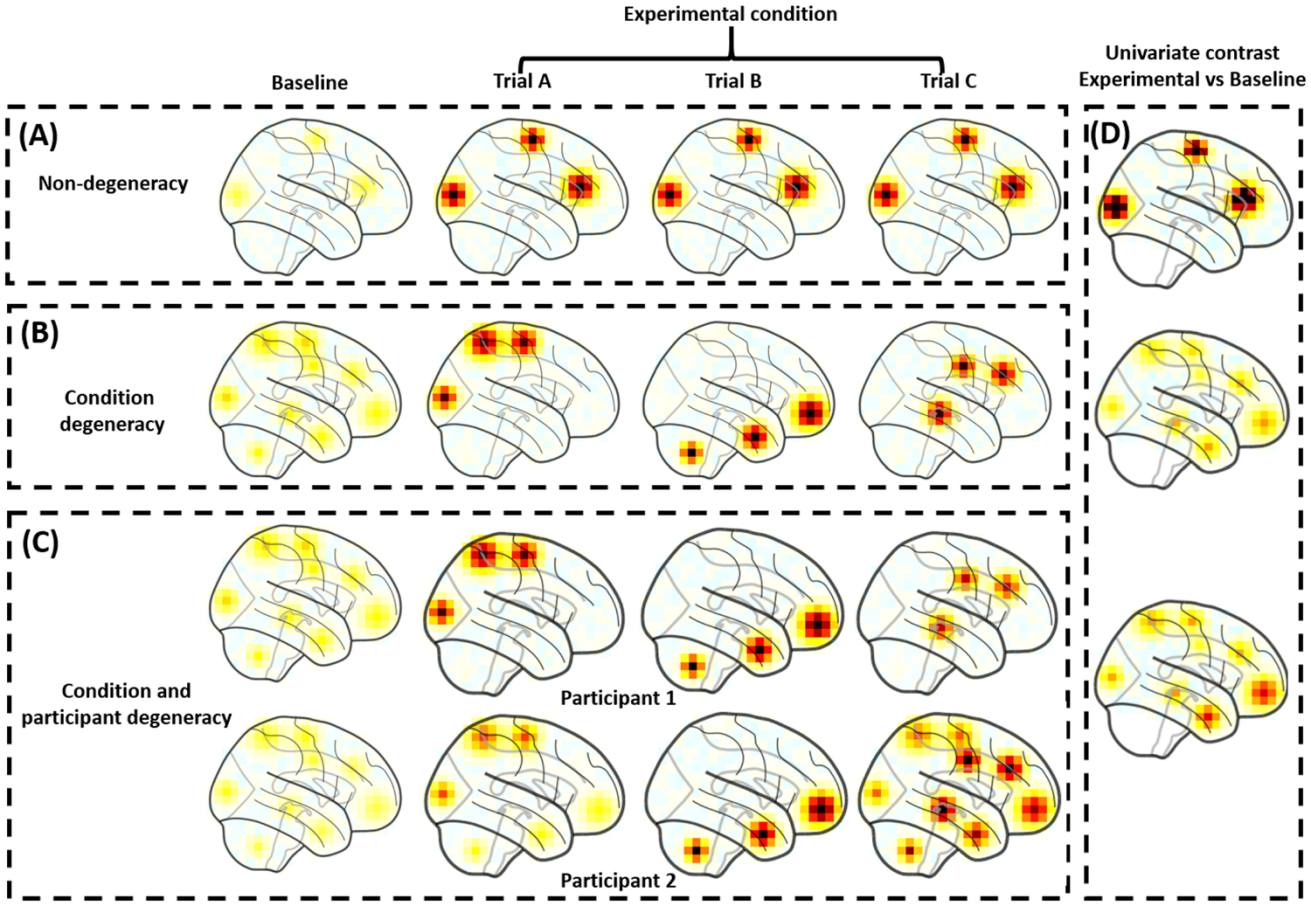
**Standard univariate analysis applied to degenerate situations**. We applied univariate analysis (right panel) to three simulated datasets (left panels), assuming a simple experimental design with a baseline condition and a task condition involving multiple trials. In an affective neuroscience task, for example, the experimental condition might be a fear condition, as designated and labeled by the experimenter, which consists of multiple trials that are thought to induce fear. **(A)** Non-degeneracy: We simulated data from a situation without degeneracy, in which a consistent set of regions are more active during the experimental condition than the baseline condition across trials (and across participants). **(B)** Condition degeneracy: Simulated data included different patterns of activation associated with different trials of the same experimental condition. **(C)** Degeneracy by condition and participant: Simulated data included different patterns of activation are associated with different trials and participants. **(D)** A traditional univariate analysis performs well in the situation without degeneracy. However, the analysis would be insensitive to the variations in the two situations involving degeneracy. Critically, with sufficient statistical power, the univariate analysis may still yield significant activations in situations B and C. However, the summary map would grossly mischaracterize the data, and the underlying data generating distribution

### Experimental Design

Rather than extensively review the various forms of degeneracy that can occur in the brain, we generated a synthetic dataset to demonstrate two aspects of degeneracy that could occur in fMRI data. There could be many reasons for degeneracy, as noted in the introduction and as we speculate upon in the discussion. The focus of this paper is to illustrate how well certain models would perform when the assumption of degeneracy by condition holds. We opt to use simulation data for two reasons: 1) the synthetic data allows us to mathematically specify the assumption of degeneracy, and 2) the synthetic data also provides a known ground truth to validate NTFA’s performance.

The synthetic dataset reflects a generic experimental framework in which participants undergo a baseline condition and an experimental condition. In this simulated experiment, participant completed eight trials total. The baseline condition has two trials and the experimental condition has six trials. Each trial contains 20 TRs. The synthetic dataset used a downsampled MNI template with a 8x8x8mm voxel size.

To offer a concrete example, in a study on fear, the baseline condition may consist of multiple trials that maintain a neutral affective state in the baseline condition and multiple trials that induce fear in the experimental condition and. In a study on working memory, there may be trials that involve low capacity demand in the baseline condition, and trials that involve high capacity demand in the experimental condition. We used the term, trial, to broadly represent trials in sequence (e.g. the first, second, ..., trial of the task), or the specific contents of a trial in a task (e.g. trials that present stimulus A, stimulus B, ..., in which each stimulus is a sampled instance from the same task). Degeneracy may occur in either case. In the simulated data, there are three trials of type A, B, C in the experimental condition and a baseline trial type in for each scenario. We varied the underlying distribution for the trial types to reflect the assumption of degeneracy.

We simulated multivariate patterns of neural activity throughout the brain by sampling from a prespecified underlying distribution. We assumed a single baseline state such that the neural activity of the baseline condition is generated from one distribution. We then modeled three hypothetical situations to reflect different assumptions of degeneracy, which are described in more detail in the subsequent section (Fig. 1). For simplicity, the analysis was performed on the synthetic dataset consisted of two participants. We simulated 20 participants and the results showed the same conclusion as results from 2 participants (See Supplementary Materials B). The simulated data used in the manuscript assumes an SNR of 8 which is well within the range for fMRI datasets (Welvaert & Rosseel (2013) found various fMRI datasets to range in SNR between 0.35 and 203. Results on simulated data over a range of lower SNR (down to 0.16) is provided in Supplementary Materials 6.

Since this paper is interested in variations in the task-related signal, the synthetic dataset is meant to resemble a denoised dataset in real life. The simulation did not include a hemodaynamic function or any nuisance related signal (e.g., head motion, white matter, CSF, etc) that might be present in the real data. We simulated the data under varying additive noise conditions to show reliability.

### Non-Degeneracy

The non-degenerate functional neuroarchitecture stipulates that experimental trials evoking a common psychological state or process share a common underlying pattern of activation. We generated simulated data to fit this assumption. We started by selecting three brain areas randomly to create a pattern of activation during experimental condition trials (Fig. 1A). We chose three areas arbitrarily to reflect the fact that the assumptions of a non-degenerate functional neuroanatomy have little to do whether the pattern of activation is localized to one area or distributed across many areas. What is important is that the same pattern of activation is assumed to occur consistently across trials and participants, and that a non-degenerate model treats variation as residual error. To capture this assumption in our synthetic data, we specified the data generating process as a unimodal distribution. This refers to one pattern of neural activity with some Gaussian distributed noise across trials and participants. The synthetic data from individual trials A, B, and C, as shown in (Fig. 1A), were sampled from this distribution. This model suggests there is a common pattern of activation across all trials that evoke fear, for example.

### Degeneracy by Condition

Degeneracy by condition refers to the existence of multiple distinct patterns of neural activation that occur across trials of the same experimental condition. Using fear as our running example, different fear induction trials may involve different patterns of brain activation (Fig. 1B). To simulate data corresponding to a degeneracy by condition model, our data generating process involved sampling from one of three different distributions. Each of the three distributions gave rise to distinct activation patterns from the others, while maintaining similar activation patterns within the distribution. In Fig. 1B, Trials A, B, and C are exemplars, with each one sampled from a different distribution. Thus, degeneracy by condition suggests that multiple distinct activation patterns may occur during trials within the same experimental condition.

### Degeneracy by Participant and Condition

For our third situation, we examined degeneracy with respect to both condition and participant. Similar to the example in the degeneracy by condition scenario, a participant would have different patterns of activation during different trials of the same experimental condition. In addition, however, the participant would also have a different pattern of neural activation than other participants, even during the same trial. For example, both participants may report experiencing the same level of fear when shown the same fear-inducing stimulus, but nevertheless show differential activation patterns.

This situation is illustrated in Fig. 1C. Two participants may be presented with the same set of trial stimuli and even have the same behavioral responses, but the underlying neural patterns may nonetheless vary. For example, in Trial A, the exemplar data from two participants share activity in dorsal areas, but one participant also shows activity in ventral areas. In Trial B, they show similar patterns of activation. In contrast, in Trial C, there are again differences between participants. Thus, our data generating procedure was designed to capture: (i) degeneracy across participants by including both participant-specific activation patterns (e.g. Trials A and C), (ii) degeneracy by condition by including variation in activation patterns across Trials A-C within a participant, and also (iii) activation patterns that are also shared across participants (e.g. Trial B).

### Univariate Analysis

We applied a standard univariate General Linear Model (GLM) to calculate a contrast between the experimental conditions and the baseline condition. We implemented the GLM in which each trial was modeled as a separate regressor, such that the model estimated a statistical map for each trial. The model then calculated a contrast on trials in the experimental condition and on trials in the baseline condition to assess which voxels showed greater activity in the experiment conditions than in the baseline condition. The average betas over two participants was presented. The model did not include nuisance regressors and were not convolved with a hemodynamic response function since the synthetic data did not include nuisance related signal and was not convolved with hemodynamic function since they were not included in the simulated dataset.

### Neural Topographic Factor Analysis (NTFA)

**Fig. 2:**
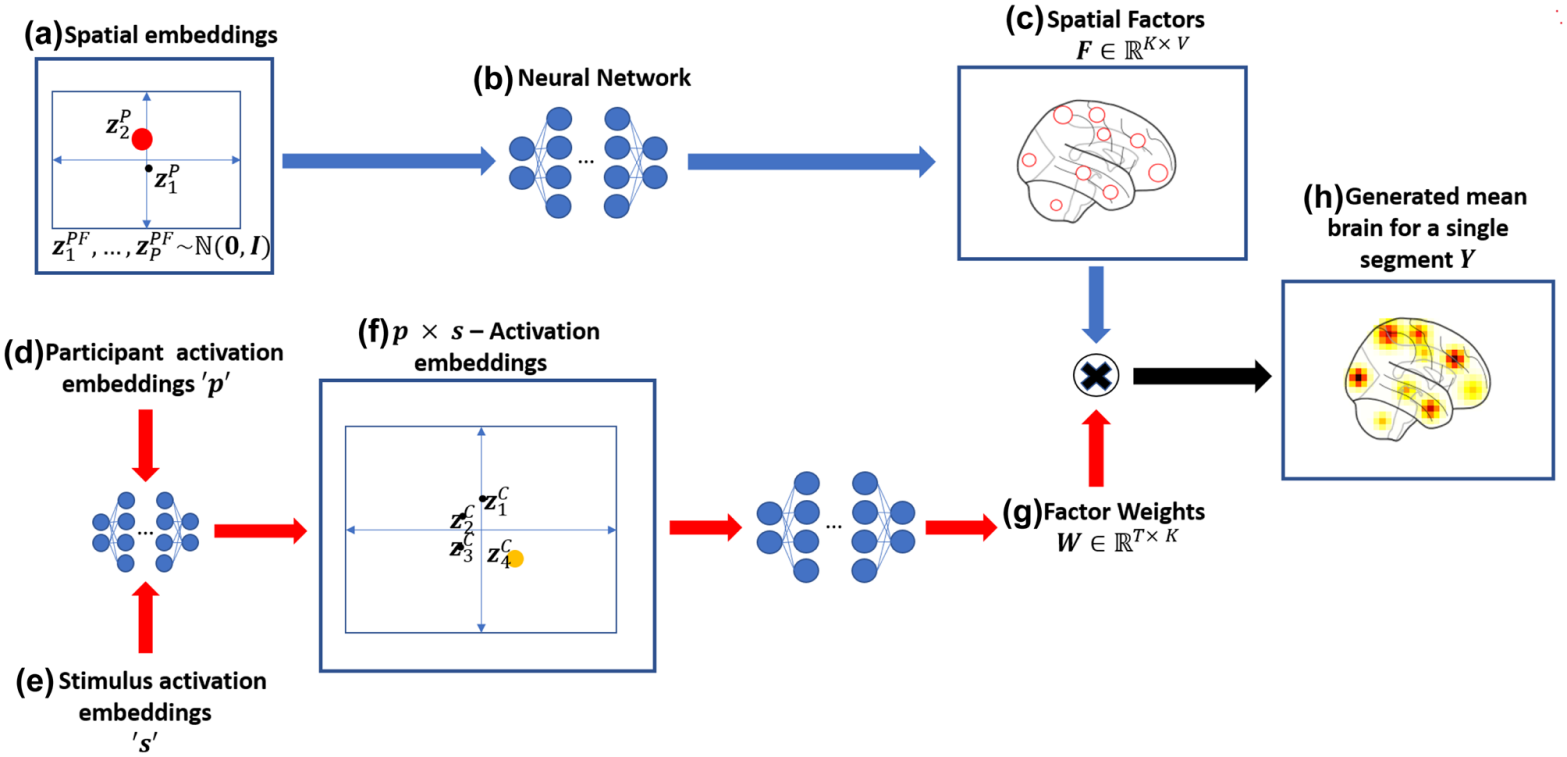
**NTFA Generative Model:** This figure describes how NTFA generates a single segment of fMRI data with *V* voxels and *T* TRs. NTFA treats a single participant-trial combination in the experiment as a segment of fMRI data such that it could model the participant and trial dependent activation without grouping participants or trials a priori. Concisely, NTFA splits this data generation into two parts, reflected by the two pathways in this figure. The first pathway, following the blue arrows, generates a participant dependent set of spatial factors. The second pathway, following the red arrows, generates the participant *and* trial dependent activation weights for these factors. The multiplication of these spatial factors and the factor weights gives us the generated fMRI segment. **(a-c) Generating spatial factors:(a)** We sample 2-dimensional spatial embeddings ($z^{{P}{F}}$) from a gaussian prior, with each dot representing a participant in the shared embedding space. For each segment we only use the spatial embedding for the participant in that segment, shown here as the red dot. **(b)** This spatial embedding is submitted to a neural network. The same neural network is shared by all spatial embeddings. The use of neural networks allows a potentially non-linear mapping between the embedding space and the variations in the spatial factors. **(c)** The neural network maps this embedding to the *K* spatial factors to represent the functional units of activation in the brain, shown as the red circles. These spatial factors are assumed to be radial basis functions parameterized by the centers and widths output by the network. Here we show these spatial factors as red circles covering two widths of the radial basis function. The Spatial Factors are mathematically denoted by a matrix *F* of size *K* x *V*. As such, the differences in the spatial embeddings reflects the variations in these spatial factors. **(d-g) Generating factor weights: (d,e)** Similar to the spatial embeddings we also sample a participant activation embedding for the same participant and trial activation embedding for the trials across task conditions corresponding to the combination. These embeddings are meant to capture overall participant and trial dependent activity respectively. **(f)** These two embeddings are then passed to a neural network to produce the corresponding $p \times s$- activation embedding. Each dot represents a unique participant and trial combination. **(g)** The activation embedding is then passed through another neural network to generate the Factor Weight matrix of *W* of size $T \times K$. The factor weights capture the activations of the spatial factors. The neural network outputs the mean and a standard deviation of activation for each factor. Each factor’s activation is then generated by sampling independently over TRs from the corresponding Gaussian distribution to create the time varying weights *W*. As such, variations in locations of these activation embeddings reflects variations in the activations of spatial factors. The embeddings provide a way to visualize high dimensional variations between brain activations for different participant-stimulus combinations. **(h)** Finally, these weights and spatial factors can be arranged in the form of two matrices $W \in \mathbb {R}^{T \times K}$ and $F \in \mathbb {R}^{K \times V}$. The matrix of spatial factors *F* and their activations *W* can be multiplied to generate data $\mathbf {Y}$ i.e. this segment of fMRI data. For a comprehensive version of this figure, see Fig. A1 in Supplementary Materials

**Fig. 3:**
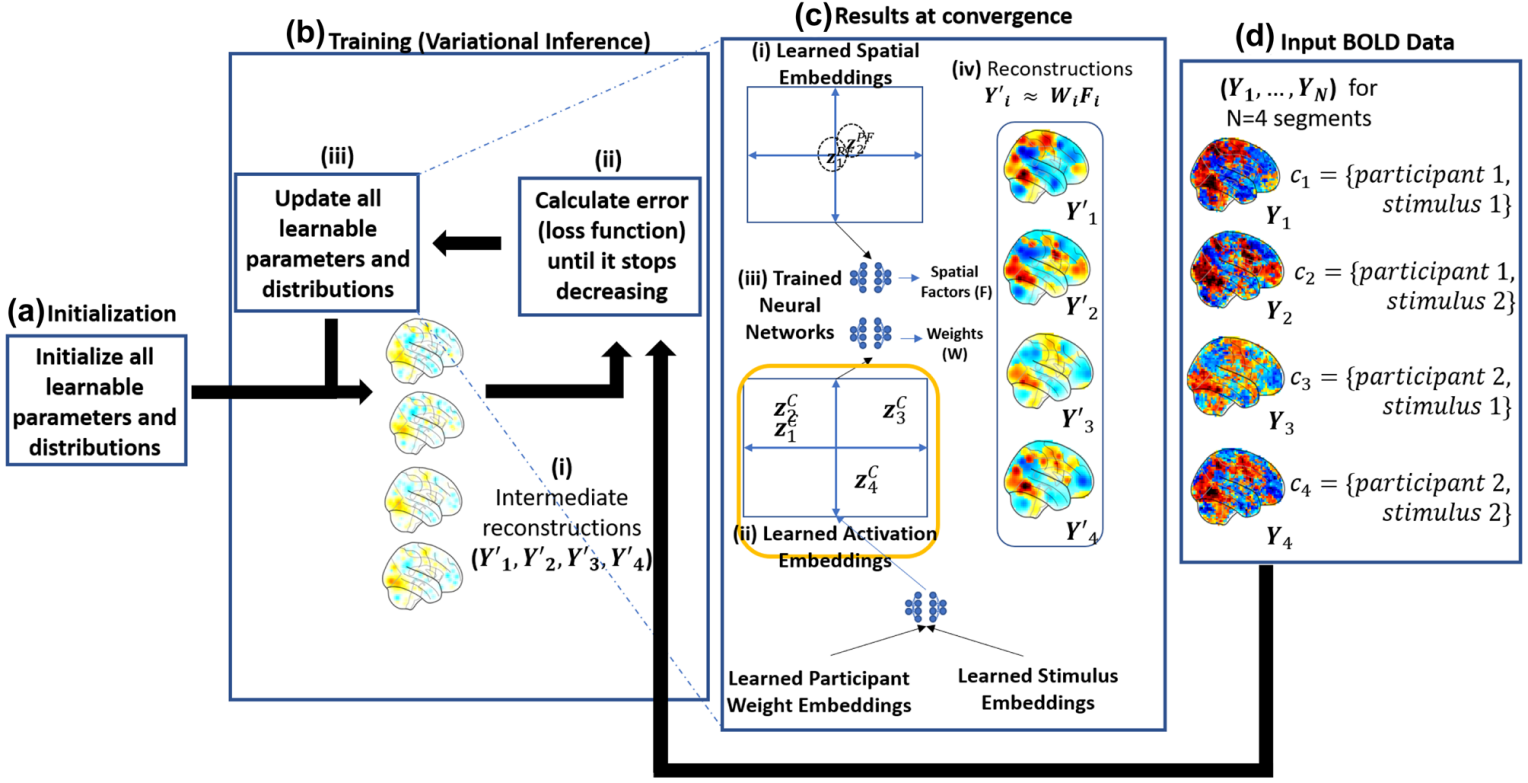
**NTFA Training using variational inference:** This figure shows the training procedure for NTFA for a hypothetical dataset that includes two participants and two stimuli for a total of four combinations. Mean brain images for the four segments can be seen in panel (**d**) where the preprocessed BOLD data is split into segments of participant-trial combinations, denoted here as $c_1, c_2, c_3$ and $c_4$ for this hypothetical example. **(a) Initialization** All parameters and distributions are initialized as specified in Supplementary Information. **(b) Training** **(b-i)** Starting of from this initialization intermediate reconstructions are generated at each step. **(b-ii)** The parameters are used iteratively to calculate the reconstructions error. The loss function is defined as the sum of reconstruction error and a regularizer (see Supplementary Information Eq. (10)for more detail). This is a consequence of using variational inference which aims to approximate the unknown posterior distributions of all the hidden variables with a set of simpler distributions, Gaussian in this case. **(b-iii)** These parameters are then updated in the direction of decreasing loss using stochastic gradient descent (SGD). The iterations are repeated until convergence that is when the loss function stops decreasing. **(c) Results at convergence** The learned parameters at convergence are represented by the embeddings. The embeddings provide a visual conclusion of variances in neural activity across different participant-trial combination. **(c-i)** The learned spatial embeddings encode the relative differences in the locations and widths of the spatial factors between participants. **(c-ii)** The learned activation embeddings are highlighted here in yellow as they are the main focus of this paper. These embeddings represent the differences in activation of the spatial factors among different participant-trial combinations. For example, in this hypothetical case the combinations 1 and 2 on the left of the plot are more similar to each other as compared to combinations 3 and 4. **(c-iii)** The three trained neural networks allow us to capture potentially nonlinear relationships between different participants’ spatial factors as well as activations for different combinations. These neural networks can also be used to generate unseen data including unseen participant-trial combinations by providing inputting appropriate embeddings. **(c-iv)** Shows the learned reconstructions that should approximate the major patterns in the input data as can be seen by side by side comparison with panel (**d**) with a limited number of spatial factors $K<< V$. For a comprehensive version of this figure see Fig. A1 in the Supplementary Materials

NTFA is a class of generative models built off of earlier topographic factor analysis (TFA) approaches for fMRI data (Manning et al., 2014b) that is designed to learn low-dimensional, visualizable embeddings from segments of data for different participant and tasks (Sennesh et al. 2019). We modify the original NTFA model such that the modified model (we will continue to referred to this modified model as NTFA, as it still consists of a neural network prior combined with a TFA likelihood) can reveal different aspects of the data, including degeneracy. Moreover, NTFA is primarily unsupervised, requiring only the participant and trial identities. We provide an overview of NTFA’s generative model and training mechanism in Figs. 2 and 3 respectively.

NTFA is designed to enable systematic comparison of functional neuroanatomy across individuals and task conditions by mapping fMRI data to low-dimensional (and visualizable) embeddings. We achieve this goal by formalizing three assumptions:

- First, we assume voxel-level data can be parsimoniously expressed as a much smaller set of functional units, which we refer to as **spatial factors**. We model these spatial factors as radial basis functions, and the activation at a given voxel as a sum of weighted contributions from these factors.
- Second, we assume that the same spatial factors exist in all participants, but their precise spatial location may vary across individuals. A set of low dimensional participant dependent **spatial embeddings** ($z^{{P}{F}}$) capture this variation. A neural network maps these embeddings to the centers (location) and widths (extent) of the spatial factors. This neural network is shared across participants. The neural network allows us to learn a possibly nonlinear mapping from the space of spatial embeddings to that of spatial factors. This is important, as the anatomical alignment literature Haxby et al. (2011); Saxe et al. (2006) makes it implausible that this relationship can be captured with a linear transformation. Similarly, sharing a single neural network among all factors and all participants allows the spatial embeddings to be commensurable between participants. A Gaussian prior on the spatial embeddings encourages them to be close to each other.
- Third, we assume that degeneracy or non-degeneracy is effectively revealed as a combination of how a single participant’s brain responds to the various trials in a task condition (i.e. participant dependent activity) and how multiple individuals might respond to a the same trial in a task condition (i.e. trial dependent activity). By combining estimates of these sources of variation, we are able to detect whether neural activity in response to the same trial varies systematically across individuals, which we refer to as participant task combinations. Similar to the approach used for the spatial embeddings, participant dependent (*p*) and trial dependent (*s*) activity is estimated across the spatial factors (through embeddings $z^{{P}{W}}$ and $z^{{S}}$ respectively) and combined through a neural network to generate $(\mathbf {p \times s})$**activation embeddings** ($z^{C}$). The use of shared neural networks here once again ensures that the low dimensional embeddings can capture non-linear effects, and makes these embeddings commensurable between different participant-task combinations.

Taken together, these spatial and activation embeddings respectively provide a low-dimensional summary of where and how individuals’ brains respond to an experiment. Critically, the activation embeddings also summarize whether such responses are shared or diverge across individuals, hereby revealing potential degeneracy. In the following subsections, we first discuss NTFA generative model in detail ("Generative Model"). We then explain the variational distribution used for the inference procedure ("Inference") and how it is initialized ("Initializing Variational Distribution"). Lastly, we explain how the NTFA deploys the variational inference procedure iteratively to estimate the distribution of the latent variables given the observed data ("Training").

#### Generative Model

The crux of NTFA’s generative model can be explained in three parts. **First** is to assume that a segment of fMRI data consisting of *T* time points and *V* voxels $Y \in \mathbb {R}^{T \times V}$ can be approximated by the matrix product of two matrices $Y \approx WF$; a matrix $F \in \mathbb {R}^{K \times V}$ that defines the spatial location of $K \ll V$ factors, with each row defining that factor’s influence over each voxel, and a matrix $W \in \mathbb {R}^{T \times K}$ defining the weight of each factor at each time instant. **Second**, the model assumes that for a given participant “*p*” in a segment, the parameters that define matrix *F* can be generated from a lower dimensional vector $z_p^{{P}{F}}$ by passing it through a trainable non-linear mapping (a neural network $\theta _{F}$ in this case). This neural network is shared across all trials and all participants, which means the factors for all participants are generated through a shared mapping and the differences in the lower dimensional vectors can be interpreted as differences in matrix *F* for participants across the experiment. **Third**, the model also assumes that given a participant-trial combination “$c = p \times s$” in a segment, the parameters that generate the matrix *W* for this segment are generated by another lower dimensional vector $z_c^{C}$ mapped through another neural network $\theta _{W}$. This neural network is also shared across trials for all possible combinations and thus the differences in the lower dimensional vectors can be interpreted as differences in the activation of the spatial factors for different participant-trial combinations. This embedding is itself the output of a neural network $\theta _{C}$ that takes as input a participant dependent embedding $z^{P}$ and a task dependent embedding $z^{S}$. In the following paragraphs we unpack this model and the underlying assumptions in more detail. This description is also summarized and presented in Supplementary Materials Fig. A1 for an example setting.

Let’s assume we want to generate fMRI data for an experiment with $n=\{1,\ldots ,N\}$ segments. Each segment *n* consists of a participant $p_n$ out of a total of *P* participants ($p_n\in \{1,\ldots , P\}$) undergoing a trial $s_n$ out of a total of *S* unique trials ($s_n\in \{1,\ldots , S\}$). This leads to every segment being defined by a combination $c_n = \{p_n,s_n\}$ of the participant identity and trial identity, where $c_n \in \{1,\ldots , C=PS\}$.

The first assumption we make is that each participant *p* has a D-dimensional spatial embedding vector $z_p^{{P}{F}}$ (Fig. 2a, Fig. S5(A)) and a participant embedding vector $z_p^{{P}}$ (Fig. 2d, Fig. S5(E)) associated with it. The participant embedding is the vectors of all participants plotted in a 2-dimensional space. The spatial embedding captures the mean and variance of the center and width for each spatial factor in the brain space. The participant embeddings captures the participant dependent response across all trials in a task condition, Similarly we assume that each trial *s* also has a separate D-dimensional trial embedding vectors $z_s ^ {S}$ (Fig. 2e, Fig. A1(F)) associated with it. We assume $D=2$ for both cases as we would like to be able to visualize these vectors. These embeddings allow us to reason about differences between participants and trials as signal rather than noise. These participant and trial embeddings then pass through a neural network $\theta _{C}$ to generate participant-trial activation embeddings $z^{C}$. These combination embeddings in turn generate through another neural netwok $\theta _W$ the parameters for the distributions of activations of the spatial factor for a given participant-trial combination.

The second assumption is that these embeddings are sampled from a standard normal prior (a gaussian distribution with zero mean and identity covariance i.e. $\mathcal {N}(0,I)$). The embeddings are assumed to lie in two *separate* 2-dimensional spaces as shown in Fig. 2a, d, e (for detailed visualization, see Fig. A1(A, E, F). Note that we will infer the distributions of each of these embeddings later, these priors serve to constrain the space in which these embeddings lie in relation to each other.

1 $$\begin{aligned} z_{p}^{P}&\sim \mathcal {N}(0,I),&z_{p}^{{P}{F}}&\sim \mathcal {N}(0,I),&z_{s}^{S}&\sim \mathcal {N}(0,I). \end{aligned}$$

The third assumption is that the participant weight embeddings $z_p^{P}$ and trial embeddings $z_s^{S}$ can be combined through a non-linear mapping (with a simple neural network) to generate the combination embedding $z_c^{C}$ for that particular participant-trial combination.

2 $$\begin{aligned} z_{c}^{C}&\leftarrow \theta _{C}(z^{P}_p,z^{S}_s), \end{aligned}$$

The fourth and the most critical assumption is that the spatial embeddings and the activation embeddings can be mapped to two matrices: a matrix of factors $F \in \mathbb {R}^{K \times V}$ and a matrix of weights $W \in \mathbb {R}^{T\times K}$ through a non-linear mapping (using neural networks). Where *V* is the number of voxels in the fMRI data and *T* is the number of time points in a segment. To realize this mapping, we assume that after sampling a participant embedding $z_p^{{P}{F}}$ using Eq. (1) it can be passed through a neural network $\theta _{F}$ that outputs four quantities. It outputs 3-dimensional means of *K* centers $\mu _p^x$ in voxel space, 3-dimensional standard deviations $\sigma _p^x$ associated with these means. Similarly it outputs 1-dimensional means of *K* log-widths $\mu _p^\rho$, and associated 1-dimensional standard deviations $\sigma _p^\rho$ (Figs. 2c, A1(C)). After generating these means and standard deviations, we assume that the *K* centers for the participant *p* i.e. $x_p^{F}$ and *K* log-widths $\rho _p^{F}$ can be sampled from Gaussian distributions with means and variances generated above (Fig. A1(D)).

3 $$\begin{aligned} x^{{F}}_p&\sim \mathcal {N}(\mu ^{x}_{p}, \sigma ^{x}_{p}),\mu ^{x}_{p}, \sigma ^{x}_{p}&\leftarrow \theta _{F}(z^{P}_p),\end{aligned}$$

4 $$\begin{aligned} \rho ^{{F}}_{p}&\sim \mathcal {N}(\mu ^{\rho }_{p}, \sigma ^{\rho }_{p}), \mu ^{\rho }_{p}, \sigma ^{\rho }_{p}&\leftarrow \theta _{F}(z^{P}_p). \end{aligned}$$

Once the centers and log-widths are sampled using Eq. (3) we can use these to define *K* spatial factors using a radial basis function. That is, each factor $f_k$ is defined as a Gaussian “blob” centered at $x_{p,k}^{F}$ with a log-width $\rho _{p,k}^{F}$. Each factor $f_k$ defines a single *V*-dimensional row of the matrix $F_p$ for participant *p* (Figs. 2d, A1(E)).

Note that the neural network $\theta _{F}$ is the same for all participants, implying that this mapping is shared across participants and for all segments. The embedding $z_p^{{P}{F}}$ once sampled for a particular participant also stays the same across all segments. These two assumptions combined indicate that there’s something common for a participant across the whole experiment, and that the embeddings for the participants can be compared with each other.

Similarly we assume after generating the activation embeddings $z_c^{C}$ using Eq. (1) for a trial *n* these can be passed through another neural network $\theta _{W}$ to generate 1-dimensional means of *K* factor weights $\mu _n^{W}$ and associated standard deviations $\sigma _n^{W}$. Then the weight for each factor can be sampled from a Gaussian distribution with the generated mean and standard deviation for each time point *t* (Figs. 2e, A1(F), (G)).

5 $$\begin{aligned} W_{n,t}&\sim \mathcal {N} \left( \mu ^{{W}}_{n}, \sigma ^{{W}}_{n} \right) ,&\mu ^{{W}}_{n}, \sigma ^{{W}}_{n}&\leftarrow \theta _{W}\left( z^{C}_c \right) . \end{aligned}$$

Once we have $W_{n,t}$ and $F_p$ for a segment our last assumption is that noisily sampling the matrix product of these two matrices generates the fMRI image at time *t* for segment *n* (Figs. 2f, A1(H)).

6 $$\begin{aligned} Y_{n,t}&\sim \mathcal {N}\big ( W_{n,t} F_{p}, \sigma ^{Y}\big ),&F_{p}&\leftarrow \text {RBF}(x^{F}_{p}, \rho ^{{F}}_{p}). \end{aligned}$$

This generative model can be summarized in the form a joint probability density over all the random variables in the model $p_\theta (Y,W,x^{F},\rho ^{F},z^{P},z^{{P}{F}},z^{S})$ which can be defined as follows:

7 $$\begin{aligned} p_\theta (Y,W,x^{F},\rho ^{F},z^{P},z^{{P}{F}},z^{S}) &= p(Y\mid W,x^{F},\rho ^{F})p_{\theta _{W}}(W \mid z^{C}\\ &= \theta _{C}(z^{P},z^{S})) p_{\theta _{F}}(x^{F},\rho ^{F}\mid z^{{P}{F}})p(z^{S})\\& \quad \ p(z^{P})p(z^{{P}{F}}) \end{aligned}$$

#### Inference

The generative model we have discussed so far and summarized in Eq. 7 describes the generation of the data. While the actual quantity of interest for us is what we can learn when we already have the data. Given data *Y* from an fMRI experiment, all the other random variables in Eq. (7) are unobserved (latent) and we’d like to learn the distribution of these latent variables given the data i.e. we are interested in the posterior distribution $p_\theta (W,x^{F},\rho ^{F},z^{P},z^{{P}{F}},z^{S}\mid Y)$. Unfortunately, learning this distribution directly is intractable since it involves multiple integrations over all possible values of all the latent variables (See: Supplementary Information Bayes Rule). Fortunately, there is a group of techniques in Machine Learning literature called Variational Inference that aim to approximate the posterior distribution with a simpler distribution defined over all the latent variables. This approximate posterior distribution is often called variational distribution and denoted as $q_\lambda$ with parameters $\lambda$.

This variational distribution is often assumed to be factorizable, in our case this means assuming a variational distribution that is the product of individual distributions defined over all the latent variables as follows:

8 $$\begin{aligned} q_{\lambda }(W, \rho ^{F}, x^{F}, z^{P},z^{{P}{F}},& z^{S}) = \prod _{n=1}^{N}\prod _{t=1}^{T} q_{ \lambda ^{W}_{n,t}}(W_{n,t})\prod _{s=1}^S q_{\lambda ^{S}_{s}}(z^{S}_s) \\&\prod _{p=1}^P q_{\lambda ^{{X}^{F}_{p}}}(x^{F}_p) \, q_{\lambda ^{\rho ^{F}_{p}}}(\rho ^{F}_p) \, q_{\lambda ^{P}_{p}}(z_p)q_{\lambda ^{{P}{F}}_{p}}(z_p^{{P}{F}}). \end{aligned}$$

where $q_{ \lambda ^{W}_{n,t}}(W_{n,t})$ approximates the posterior distribution of factor weights for trial *n* and time point *t*. $q_{\lambda ^{S}_{s}}(z^{S}_s)$ approximates the posterior distribution of trial embedding for trial *s*. $q_{\lambda ^{{X}^{F}_{p}}}(x^{F}_p)$ approximates the posterior distribution of factor centers for participant *p*, while $q_{\lambda ^{\rho ^{F}_{p}}}(\rho ^{F}_p)$ does the same for factor log-widths. $q_{\lambda ^{P}_{p}}(z_p)$ approximates the posterior distribution for the participant embedding for participant *p* and $q_{\lambda ^{{P}{F}}_{p}}(z_p^{{P}{F}})$ does the same for participant facto embedding .

Once we have defined the variational distribution in Eq. (8) the next step is to learn the parameters $\lambda = \{\lambda ^{W}, \lambda ^{S},\lambda ^{X},\lambda ^\rho ,\lambda ^{P},\lambda ^{{P}{F}} \}$ of this distribution and the neural network parameters $\theta = {\theta _{W},\theta _{F}, \theta _{C}}$ such that it comes as close as possible to the true posterior $p_\theta (W,x^{F},\rho ^{F},z^{P},z^{{P}{F}},z^{S}\mid Y)$. Once again using well known derivations (detailed in Supplementary Materials) this can be done without knowing the actual posterior distribution by instead maximizing the following objective with respect to $\lambda$ and $\theta$:

9 $$\begin{aligned} \mathcal {L}(\theta , \lambda ) = \mathbb {E}_{q} \left[ \log \frac{p_{\theta }(Y, W, x^{F}, \rho ^{F}, z^{P},z^{{P}{F}}, z^{S})}{q_{\lambda }(W, x^{F}, \rho ^{F}, z^{P},z^{{P}{F}}, z^{S})} \right] \end{aligned}$$

The right hand side of this equation can be split into two parts:

10 $$\begin{aligned} \mathcal {L}(\theta , \lambda ) = \underbrace{\mathbb {E}_{q}[\log p(Y|W,x^{F},\rho ^{F})]}_\text {negative of reconstruction error~} - KL (q_{\lambda }(W, x^{F}, \rho ^{F}, z^{P},z^{{P}{F}}, z^{S}) \mid \mid p_{\theta }(W, x^{F}, \rho ^{F}, z^{P},z^{{P}{F}}, z^{S})) \end{aligned}$$

Since $p(Y|W,x^{F},\rho ^{F})$ is a Gaussian distribution, the first time on the right is equivalent to the negative of the expected reconstruction error between the observed data and the data reconstructed from the samples from the variational distribution $q_\lambda$. The second term is a regularizer term that measures how similar the variational distribution is to the prior distribution. Maximizing this objective with respect to $\lambda ,\theta$ then equates to minimizing the reconstruction error as well as making sure that the priors and the variational distribution become similar.

This objective can be optimized using black-box methods provided by available libraries such as Probabilistic Torch (Siddharth et al., 2017). Broadly this optimization proceeds in two steps, the first is to initialize the parameters of the variational distribution $q_\lambda$ and second is to sample from this *q*, calculate the objective (10) and then to iteratively update all parameters of *q* in such a way that the objective is expected to increase until it stops increasing. We now discuss these two steps in the following paragraphs:

#### Initializing Variational Distribution

All distributions are assumed to be gaussian, owing to the universality of gaussian distributions and the ease of sampling and optimizing objective (10) when using gaussian distributions. This is also a fairly established standard practice in variational inference. Below we provide a list of how the means and variances of these gaussian distributions are initialized.

- The variational distributions over the participant embeddings $q_{\lambda ^{P}_{p}}(z_p)$ $q_{\lambda ^{{P}{F}}_{p}}(z_p)$ for a participant *p* and trial embeddings $q_{\lambda ^{S}_{s}}(z_s)$ for a trial *c* are both initialized with a zero mean and unit variance. i.e. a standard normal. When we learn these distributions, we will not only learn a point estimate for these embeddings, but also an estimate of our uncertainty about the location of each embedding. The same initialization is used for all participants, and all combinations.
- The means of variational distribution over the centers of the factors $q_{\lambda ^{{X}^{F}_{p}}}(x^{F}_p)$ and the means of variational distribution over factor log-widths $q_{\lambda ^{\rho ^{F}_{p}}}(\rho ^{F}_p)$ can be initialized in two ways suggested by Manning et al. (2014b): **1.** The means of centers can be initialized by performing k-Means clustering on the voxel locations using number of factors *K* as number of clusters. The centers of the resulting clusters can then be used to initialize the means of factor centers. Each voxel is then labeled by the center closest to it and the variance of each cluster is used to initialize the mean of the width of each factor. **2.** By hotspot initialization, While this process is described in more detail in the Supplementary Material it involves placing the initial factor centers one by one at the peak of average fMRI image calculated from the whole dataset, solving a least square problem to approximate the width of that factor, subtracting this factor from the mean image and choosing the next peak as the next center until all factors have been initialized. The hotspot initialization works well for smaller number of factors for example when dealing with simulated data. A standard deviation of 1 is used to initialize the standard deviation of the variational distributions for factor centers. For factor widths, the standard deviation is initialized as the standard deviation of widths for all factors.
- The means of variational distribution for weights $q_{ \lambda ^{W}_{n,t}}(W_{n,t})$ are initialized by constructing the initial spatial factors using the centers and log-widths from the previous step (using a radial basis function), and then solving an ordinary least squares (OLS) problem. The OLS problem uses the average brain image computed across the whole dataset, and tries to learn the weights of the initial factors such that the weights and the factors combine can approximate this average image. The resulting weights are then used as mean of variational distributions for weights for all segments *n* and time points *t*. Once again the standard deviation is initialized to 1.

#### Training

Once the variational distribution $q_\lambda$ has been initialized, we can sample from this distribution and approximate the objective (10). At first iteration we sample the variables $W,x^{F},\rho ^{F},z^{P},z^{{P}{F}},z^{S}$ from the initialized distributions for factor weights, factor centers, factor log-widths, participant embeddings and combination embeddings. This and the initial (random) weights of the neural networks are used to calculate the objective (10). This is equivalent to calculating the reconstruction error between the input data and the data reconstructed from the the sampled factor weights and spatial factors, and a regularizer term that calculates the KL divergence between model prior distribution and the variational distribution. The parameters of the variational distribution $\lambda$ and the parameters of the neural networks $\theta$ are then updated using stochastic gradient descent in a direction that improves the expected reconstruction error in the next iteration and also makes the model priors and the variational distribution more similar. This process ensures that variational distribution is updated in such a way that samples from it can reconstruct the data well, at the same time the neural network parameters are updated in such a way that samples generated from the model will be more and more similar to the samples from the variational distribution. This process is repeated until convergence which is achieved when the value of the objective function in Eq. (10) stops changing for successive iterations. Once convergence is achieved we can analyze the posterior distributions of the participant embeddings and the combination embeddings by visualizing their means and standard deviation. A detailed example of this is shown in Supplementary Materials Fig. A2. We can also visualize the reconstructions by combining the posterior estimates of weights and factors. Similarly, at this point the neural network $\theta _{C}$ is trained to generate combinations which in turn can generate average reconstructions for a segment through the trained neural networks $\theta _{W},\theta _{F}$ and can also be used to generate data similar to the training data by providing embeddings as input.

## Results

### Univariate Results

The GLM resembles a supervised analytical approach insofar as experimenters must specify beforehand the regressors in the model. In so doing, experimenters must make assumptions about how trials are nested into conditions. We evaluate how a standard univariate analysis using a GLM performs on the three synthetic datasets. In our example experiment, Trials A, B, and C, would all be modeled with a single regressor since they belong to the same experimental condition. A non-degenerate functional architecture was quantified as having the same data generating mechanisms across all trials in the experimental condition in the first synthetic dataset. The GLM has a single regressor for Trials A, B, and C, sharing the same assumptions as the data generating process. Applying the GLM to this synthetic data shows that it perfectly suits the non-degenerate functional neuroanatomy (Fig. 1D top).

In the situation of degeneracy by condition, there are multiple underlying data generating processes across different trials in the experimental condition. a standard univariate analysis does not perform well. The univariate activation result (Fig. 1D middle) appears as an amalgam of the three data generating distributions. Without knowledge of the actual data generating process, experimenters would again model the data using a single regressor for Trials A, B, and C – even though the underlying distributions are heterogeneous. In other words, the standard GLM requires the experimenter to make assumptions about how trials are organized into experimental conditions, with one of those assumptions being the absence of degeneracy. As a result, the GLM precludes the ability to test whether there is, or is not, a degenerate relationship. Even when the ground truth (i.e. the underlying generative process) exhibits degeneracy by condition, the standard univariate analysis may still produce seemingly “reliable” findings (i.e. significant and reproducible findings with enough participants). However, the resulting pattern of activation in Fig. 1D (middle) would not accurately capture the actual data generating process. Consequently, it could lead to a mistaken, but statistically “reliable”, conclusion about the relationship between neural activity and the experimental condition.

Lastly, in the situation of degeneracy by Participant and Condition, the data generating process varies across participants and experimental condition. The standard univariate approach are insensitive to variations across trials, compounded by degeneracy across individuals (Fig. 1D bottom). It treats the systematic variation in activation patterns across trials and participants as error. Though it may produce reliable findings with sufficient power, it would result in a diffuse pattern of activation that is not representative of the data generating process.

Critically, the later two synthetic datasets highlight important assumption of standard univariate analyses. The analytical procedure of a GLM involves stages such that the outputs of the trial- and subject-level analyses are inputs to the group-level analyses. This sequence of analyses assumes a nested data structure in which trials of an experimental condition within one participant’s data and each participant from their group are from one normal distribution. This assumption is valid under a non-degeneracy functional neuroanatomy (Fig. 1A), but could preclude the ability to examine degeneracy in the functional neuroanatomy (eg., Fig. 1B, C). Instead of applying the same first level model to all participants, a more appropriate model would fit the run and participant level simultaneously without assuming this nested structure.

The study demonstrates the consequences of applying widely used univariate analyses (Monti, 2011) to synthetic data that exhibit degeneracy. The results illustrate the pitfalls of using traditional univariate analyses in terms of capturing degeneracy. In light of the shortcomings of the standard univariate analysis, there is a need for models that can uncover degeneracy when it is present in the data. In the next session, we applied NTFA to the synthetic dataset to test the utility of NTFA (Sennesh et al., 2019) in addressing this complexity.

### NTFA Results

**Fig. 4:**
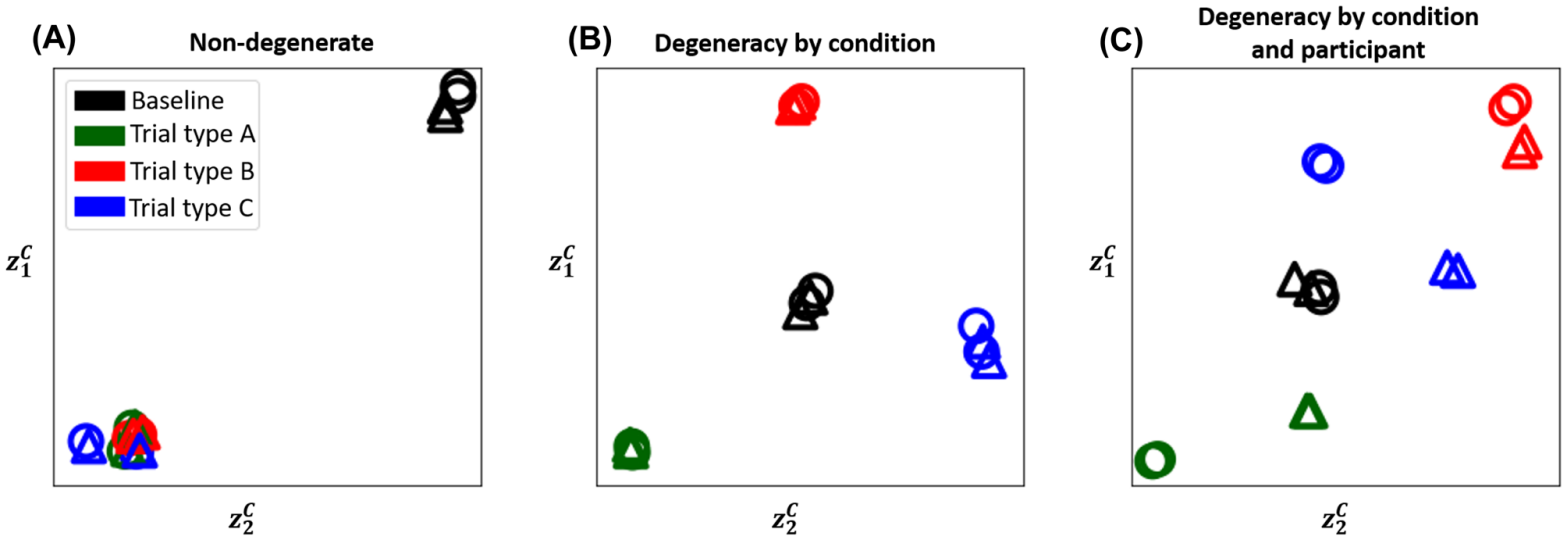
**Inferred activation embeddings:** The activation embeddings learned from NTFA for the three scenarios depicted in Fig. 1 are shown here. NTFA was trained in an unsupervised manner and labels and colors are overlaid only for visualization and interpretation purposes. Each point represents a unique participant-trial combination. The colors correspond to trials as shown in the legend. Circles represent participant 1 and triangles represent participant 2. **(a) Non-degenerate:** The embeddings suggest there is no degeneracy, with combinations for all three experimental condition trials grouping together and away from the baseline combinations. **(b) Degeneracy by condition:** The embeddings suggest degeneracy in brain response based on trials, as the combination embeddings for each trial form a cluster of its own away from other clusters and away from baseline. There are no participant driven differences suggesting no degeneracy by participants. **(c) Degeneracy by condition and participants:** The embeddings here suggest degeneracy by both trials as well as participants, with the combinations forming groups of their own based on not just trials, but also splitting up by participants in case of Trial A and Trial C

For the simulated data from the three models discussed above, we can observe the inferred activation embeddings for each of the three scenarios and see if they arrange themselves in the expected group structure for each scenario:

- **Non-degenerate**: For the non-degenerate scenario discussed in "Non-Degeneracy", we would expect the participant-trial activation embeddings to broadly fall in just two clusters: one for baseline and the other for the experimental condition. Figure 4A shows that the embeddings learned from NTFA indeed fall into two clusters.
- **Degeneracy by condition**: In the scenario discussed in "Degeneracy by Condition", the activation embeddings are expected to fall in four distinct clusters: one for the baseline, and one each for the three underlying degeneracy modes. These will correspond to the differences in the three trials. Figure 4B shows that is indeed the case for the learned embeddings on this data.
- **Degeneracy by condition and participant**: In the scenario discussed in "Degeneracy by Participant and Condition", the activation embeddings can be expected not only to group by trial, but also to split up by participants, with trials A and C revealing the degeneracy by condition and participants. Figure 4C shows precisely this expected behaviour.

We also trained NTFA on simulated data using 20 participants These results along with results for simulated data at various additive noise levels is provided in Appendix B. The inferred embeddings in these cases also have the same expected structure as presented here for a range of SNR. Embeddings only lose structure when SNR is lowered to 0.16, which is a very aggressive level of added noise, for context Welvaert & Rosseel (2013) found typical SNR to be between 0.35*and*203 for fMRI.

## Discussion

Recent work in computational biology and functional neuroanatomy suggests that the brain may have multiple solutions, or degenerate neural pathways, when trying to solve a given task. However, current analytical methods are not optimized to capture such degeneracy. Here, we advanced a novel computational approach, NTFA, to address this issue. NTFA is a generative model that learns a low-dimensional space of embeddings from the temporal and spatial variation of fMRI data. The embeddings yield a visualizable representation of the latent variations in functional activity across trials and participants. The distribution of these embeddings can provide useful information for researchers to assess whether the data generating mechanism is degenerate or non-degenerate with respect to trial conditions and participants.

NTFA is designed to capture the underlying variations that depends on the combination of the tasks and participants. The output of NTFA provides a visual representation of these variations. The core features of NTFA are designed to facilitate comparison across task and participants. Related to NTFA, there are other models that also use latent factorization methods to analyze fMRI data, however, they are not currently equipped for modeling degeneracy with respect to task conditions and participants. For example, hyper alignment (Haxby et al., 2011) and dictionary learning methods (Mensch et al., 2017; Iqbal et al., 2018) focus on characterizing subject-specific spatial variations (e.g., the precise location of the fusiform face area differ across individuals (Saxe & Kanwisher, 2003; Saxe et al., 2006)). Standard factor analysis, such as principal component analysis (Pearson, 1901) focuses on identifying components that best explain the overall covariance in the structure of the data, as Sennesh et al. (2019) demonstrate this linear projection of the data fails to capture complex and potentially nonlinear underlying structures in the data and thus would not be suitable to investigate degeneracy. Like other methods in the family of topographic factor analysis (Manning et al., 2014a; Manning et al., 2014b; Gershman et al., 2011), NTFA is useful in revealing the hidden structures in the fMRI data. However, the methods in this family differ in their assumption about the hidden structures, such as whether neural activity in the same task condition share the same structure. These methods may be able to identify degeneracy in simple scenarios where different groups of participants show clearly different patterns for the same task. For more complicated scenarios of degeneracy e.g. where the degeneracy arises from how different participants interact with different tasks in potentially non-linear ways, models that don’t explicitly account for such variance will likely fail to capture this degeneracy. NTFA learns embeddings of the unique combination of trials and participants such that it does not impose a shared structure across participants or task conditions. Of note, NTFA is flexible in its implementation. If researchers preferred to label their trials as belonging to specific task conditions, or participants as belonging to specific groups, NTFA can accommodate these assumptions and develop a generative model with these assumptions built in (e.g. for more direct comparison with other approaches). NTFA’s other features may also be useful to the community. For example, NTFA explicitly models variation in the locations, sizes, and magnitudes of activation, whereas the vast majority of studies using univariate analysis of fMRI data focus only on activation magnitudes.

NTFA is, of course, not without some limitations, one of which is determining whether learned embeddings are modeling functionally meaningful signal or simply noise. It is commonly assumed that residual noise is randomly distributed error once all sources of “systematic noise” are accounted for, for example by using aggressive denoising procedures to remove spurious signals related to motion, signal drift, physiological noise artifacts, scanner artifacts, etc. We embedded this assumption in our simulated data and note that interpretation of our algorithm’s performance on real datasets will similarly benefit from denoising procedures. To examine how noise may influence model performance, we also introduced different levels of noise into our simulations and showed how NTFA’s affected by SNR ("Conclusion"). Although much variation in fMRI data across time/trials (and across participants) is noise and should be discarded, that does not mean that all (or even most) variation unaccounted for by standard modeling approaches is necessarily noise. Here, we suggest that there is good reason to think that such variation might be structured and functionally meaningful (as described next), that historical approaches are insensitive to such variation unless it aligns with a narrow range of *a priori* hypotheses, and that NTFA is a technique that is designed to sift potentially interpretable, structured variation from random noise.

While our primary aim is constrained to establishing and validating our model using simulations, highlighting some relevant research findings may point to useful future directions in which to develop applications for NTFA. In general, it is well-known that psychological tasks are not “process pure” (Jacoby, 1991; Surprenant & Neath, 2013). A given task may involve a variety of different cognitive processes, neural pathways and/or strategies, which may shift and change over time and trials. Indeed, carefully constructed experiments have found results consistent with degeneracy even when using more traditional analytical tools. For example, dissociable neurocognitive memory systems can be used to complete the same overt memory task (Morgan et al., 2020; Zeithamova & Maddox, 2006; Knowlton & Squire, 1993; Casale & Ashby, 2008). When one system is compromised due to brain damage, other systems may be utilized to nonetheless complete the task at hand (Poldrack & Packard, 2003; White & McDonald, 2002; Price & Friston, 2002). An increasing number of findings suggest that the brain is likely to offer multiple solutions in other domains too, such as in social cognition (Lieberman et al., 2004; Amodio, 2019) and emotion (Satpute & Lindquist, 2019; Azari et al., 2020). NTFA may also be of particular relevance for translational research. Emerging work suggests that distinct neuropathologies may underlie a common clinical phenotype (Fried, 2017). For example, research on depression suggests that there may be many different neuropathologies that give rise to depressive symptoms (Beijers et al., 2019; Müller et al., 2017; Price et al., 2017a, b). Indeed, the call for “precision medicine” reflects a general failure of more traditional, non-degenerate theoretical models and rigid analytical approaches to account for heterogeneity in the underlying neural causes of mental health. A systematic evaluation of this variance is a critical step towards enabling precision medicine approaches in fMRI, in which neuroimaging studies have the potential to significantly advance diagnosis and treatment (Fonseka et al., 2018).

Despite these notable empirical examples, more often than not researchers assume that a given task involves a core set of processes that are shared across trials and participants. This may be because more traditional theoretical models in cognitive neuroscience rarely postulate degeneracy in functional neuroanatomy. However, more recent, predictive processing models of the brain suggest that degeneracy is likely to be common in mind-brain mapping (Sajid et al., 2020; Hutchinson & Barrett, 2019; Lee et al., 2021). Another reason that researchers tend to assume a non-degenerate functional neuroanatomy is because it has been analytically challenging to not make this assumption. By addressing this analytical gap, NTFA offers new opportunities to model structured variance in fMRI data with a degree of independence from our own preconceived ideas of how this variance ought to be structured, and the opportunity to discover and model degeneracy in functional neuroanatomy.

## Conclusion

Degeneracy is a ubiquitous phenomenon in complex biology systems but has yet to be systematically modeled in human neuroimaging studies. To address the analytical gap in modeling degeneracy in functional neuraoanatomy, we proposed and validated the utility of NTFA in this regard. The current study compared the performance of NTFA and standard analytical approach on synthetically generated datasets that depicted neural model of non-degeneracy, degeneracy by condition, and degeneracy by condition and by participant. The standard univariate analysis and NTFA both detected the activation pattern in the non-degenerate model that one set of brain region consistently showed higher activation for the task than the baseline in all subjects. When there was more heterogeneity of the neural activity across condition and subjects, the univariate analysis failed to capture the effect. The NTFA was able to recover participants and stimuli embeddings that distinguish different participants and different stimuli types. It provides a first step towards formally characterizing degeneracy.

## Information Sharing Statement

The code used in this manuscript can be found at https://github.com/zqkhan/ntfa_degeneracy. The data generated and analyzed in this manuscript, and the code used to generate the data can be downloaded at https://www.dropbox.com/sh/pzbmgdnsojm0abb/AAAP6XIaAuq1Ih71eGUpKNrta?dl=0

## Electronic Supplementary Material

Below is the link to the electronic supplementary material.

Supplementary file1 (PDF 2.0 mb)
