# Supplementary material for: A Computational Neural Model for Mapping Degenerate Neural Architectures
Source: Neuroinformatics. 2022 Mar 29;20(4):965–79. doi: 10.1007/s12021-022-09580-9 (PMC9588472; doi:10.1007/s12021-022-09580-9)
Supplement: Supplementary file 1 — Supplementary file1 (PDF 2.0 mb) [file 12021_2022_9580_MOESM1_ESM.pdf]

Supplementary Materials

A Neural Topographic Factor Analysis (NTFA)

Figure A.1 and Figure A.2 are provided here as detailed counterparts to Figures 2 and 3.

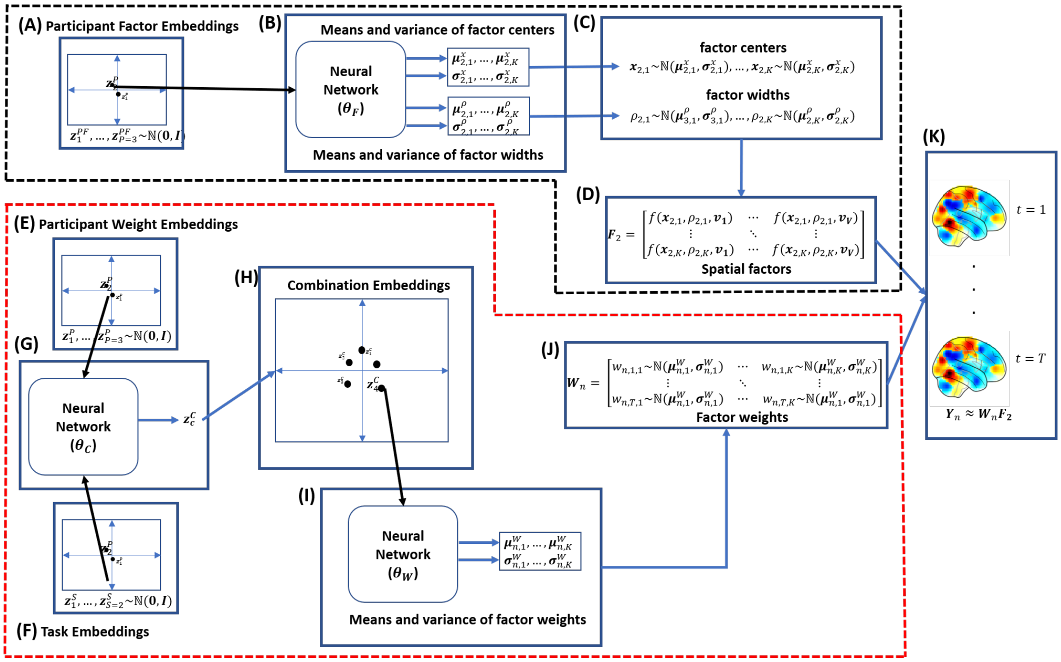

Figure A.1: **Generative Model of NTFA.** The figure illustrates NTFA's generative model. For ease of visualization we consider the case with $P = 3$ participants, $S = 2$ unique trials and therefore $C = 6$ unique participant-trial combinations. The generative model can be divided into two main parts. The first part outlined in black dashed lines generates the spatial factors (F). The second part (inside red dashed outline) generates the weights (W) of these factors. Matrix product of W and F gives us the data Y. **(A)** The participant factor embeddings $z_1^{\text{PF}}, z_2^{\text{PF}}, z_3^{\text{PF}}$ are sampled from a standard Gaussian prior. **(B)** The neural network θ_{F} takes that participant's embedding $\mathbf{z}_2^{\text{P}}\text{F}$ as an input and outputs a set of parameters; the means and variances of centers $\mathbf{x}$ and widths ρ for each of K spatial factors. **(C)** For each factor, the centers and widths are then sampled from a gaussian distribution with mean and variances from the output of the neural network θ_{F}. **(D)** The spatial factors for this participant are now constructed from these sampled factor centers and factor widths by using radial basis functions $rbf(x, \rho, v)$. Each row of the factor matrix F_3 describes one spatial factor. The rbf function takes as input the center of a factor x, the width ρ and a voxel location v to calculate the spatial coverage of the factor at voxel location v. **(E)** Participant weight embeddings $z_1^{\text{P}}, z_2^{\text{P}}, z_3^{\text{P}}$ are also sampled from a standard Gaussian prior. **(F)** Similarly, trial embeddings $z_1^{\text{S}}, z_2^{\text{S}}$ are also sampled from a standard Gaussian prior. **(G)** The neural network θ_{C} takes as input a trial embedding and a participant embedding sampled previously and generates the corresponding combination embedding. **(H)** Repeating the process in part (G) for all possible participant-trial pairs results in the combination embeddings shown here. **(I)** The neural network θ_{W} takes the appropriate embedding z_4^{C} as input and outputs the means and variances for the weights of the spatial factors. **(J)** The factor weights at each time instant $t = 1, \ldots, T$ are then calculated by sampling from a gaussian distribution with the appropriate mean and variance for that factor. **(K)** The fMRI data Y_n is then assumed to be approximately a matrix product of W_n and F_3 with additive gaussian noise σ^Y. This same process can be repeated for any segment for other participants p and combinations c to create a whole dataset with N segments.

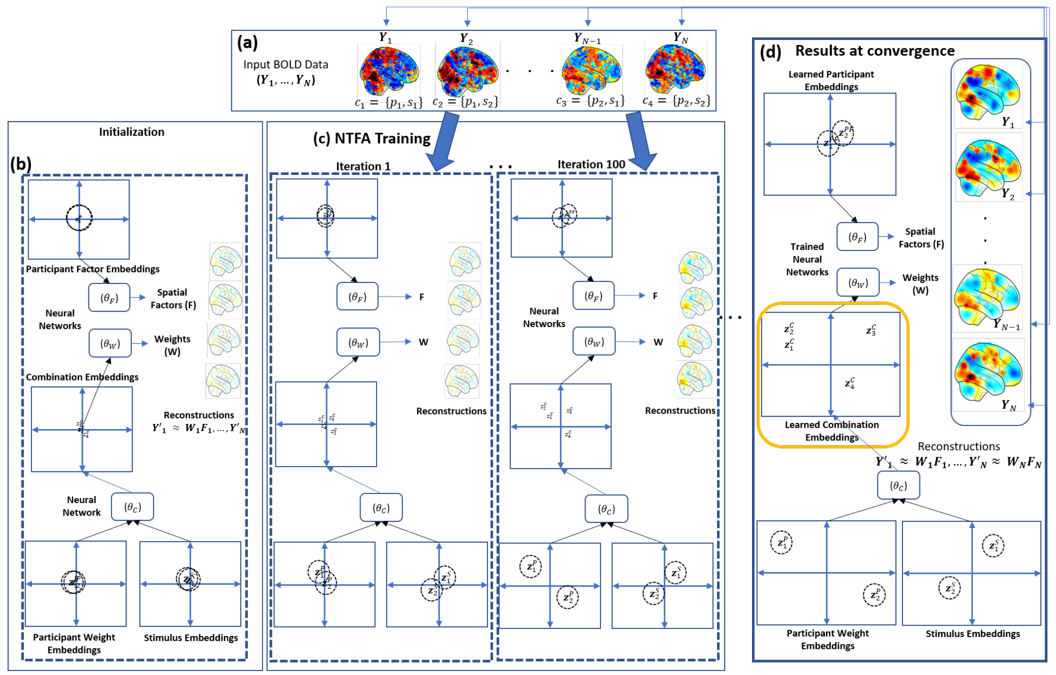

Figure A.2: **NTFA Training.** **(a)** During training NTFA takes as input an fMRI dataset with N trials, $Y_1, \dots, Y_N$, for illustration purposes this dataset is assumed to have $P = 2$ participants and $S = 2$ unique trials, which implies $C = 4$ unique participant-trial combinations, note that a combination can occur multiple times in an experiment. **(b)** Variational distributions for all the latent variables are initialized as explained in Section 2.6.3. **(c)** The model trains by iteratively optimizing the variational distributions for the participant factor embeddings (z^{P}), participant weight embeddings ((z^{PF})) and trial embeddings (z^{S}) as well as parameters of spatial factors and weights, and the three neural networks ($\theta_{\mathrm{F}}, \theta_{\mathrm{W}}$, θ_{C}). θ_{C} takes as input the participant weight embeddings and the trial embeddings to generate combination embeddings z^{C}. θ_{F} and θ_{W} generate the parameters of spatial factors and weights such that at each iteration the model is better able to reconstruct the original fMRI data. We show here the first iteration and 100th iteration. As 100th iteration the model is starting to learn the relative locations of the embeddings and the reconstructions are looking sharper as compared to iteration 1. **(d)** Once the training converges, the latent variables stop changing. The participant factor embeddings are still close to each other with overlap, suggesting the participants don't differ significantly in terms of the spatial factors. The participant weight embeddings are in clearly different regions of the space with no overlap, suggesting significant differences in the factor weights for the two participants. Similarly, the trial embeddings also suggest a significant difference between the two trials in terms of evoked response. The combination embeddings (highlighted in yellow) capture the relative differences in participants' brain response to each task. In this case, they suggest the first two combinations c_1, c_2 are more similar to each other as compared to c_3, c_4. c_3 and c_4 also appear to be different from each other. Lastly, the reconstructions at this point are reasonable approximations of the input data.

B RESULTS WITH NOISE AND MULTIPLE SUBJECTS

We provide results for increased number of subjects (20 as opposed to 2 in the main text) and the recovered embeddings are still structured as expected. See Figure B.1. Results on the same using univariate analysis can be seen in Figure B.

In all our simulations we add Gaussian voxel noise on top of our simulated data. Gaussian noise has been used to approximate system noise that depends on Signal to Noise Ratio (SNR) value in fMRI data (Ellis et al., 2020; Welvaert & Rosseel, 2013). In the results we present in the main text our mean signal magnitude is 0.8 and we add Gaussian noise with standard deviation of 0.1 each voxel, with an SNR of 8. To explore robustness of our method to noise we also increase this noise standard deviation by factors of 2, 5, 8, 10, 20, and 50. This results in SNR dropping to $4, 2, 1, 0.8, 0.4, 0.16$, Note that this is within the typical range of SNR in real datasets, which can vary between 0.35 to 203 (Welvaert & Rosseel, 2013). As expected the recovered embeddings become less and less ”meaningful” as we increase noise, specially for the more complicated scenarios 2 and 3. But even at noise with SNR of 0.4 the embedding structure still resembles what is expected. At SNR of 0.16 the noise seems to take over and the method fails to recover meaningful embeddings in scenarios 2 and 3. See Figure B.3.

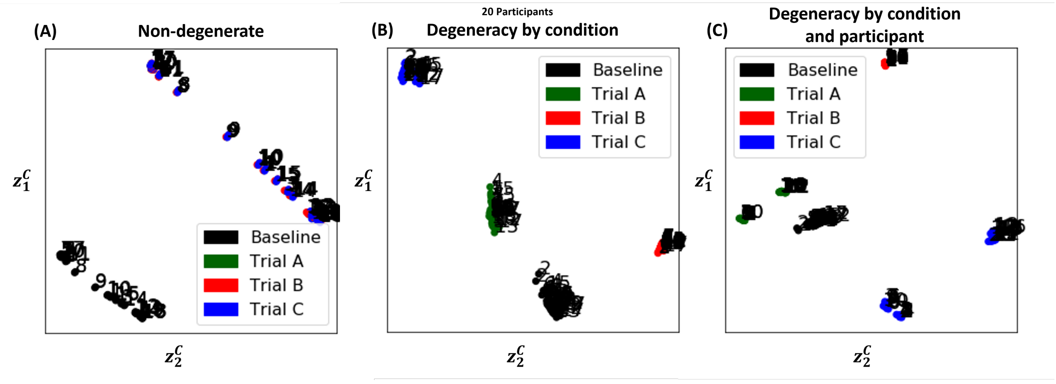

Figure B.1: Inferred embeddings from NTFA on the synthetic datasets with 20 participants. The visualizations become crowded but the recovered embedding structure is similar to the embeddings of 2 participants.

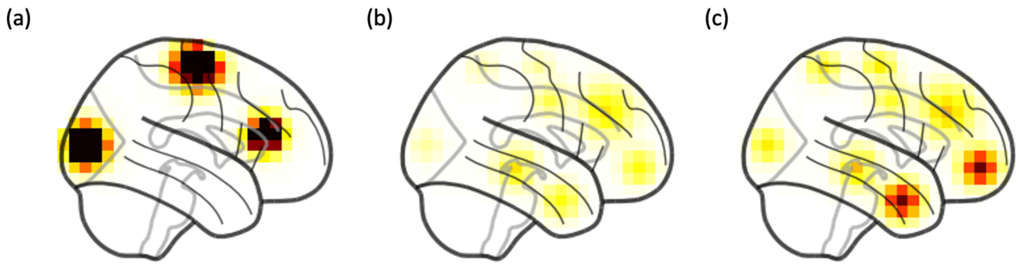

Figure B.2: Results from the univariate analysis on the synthetic datasets with 20 participants.

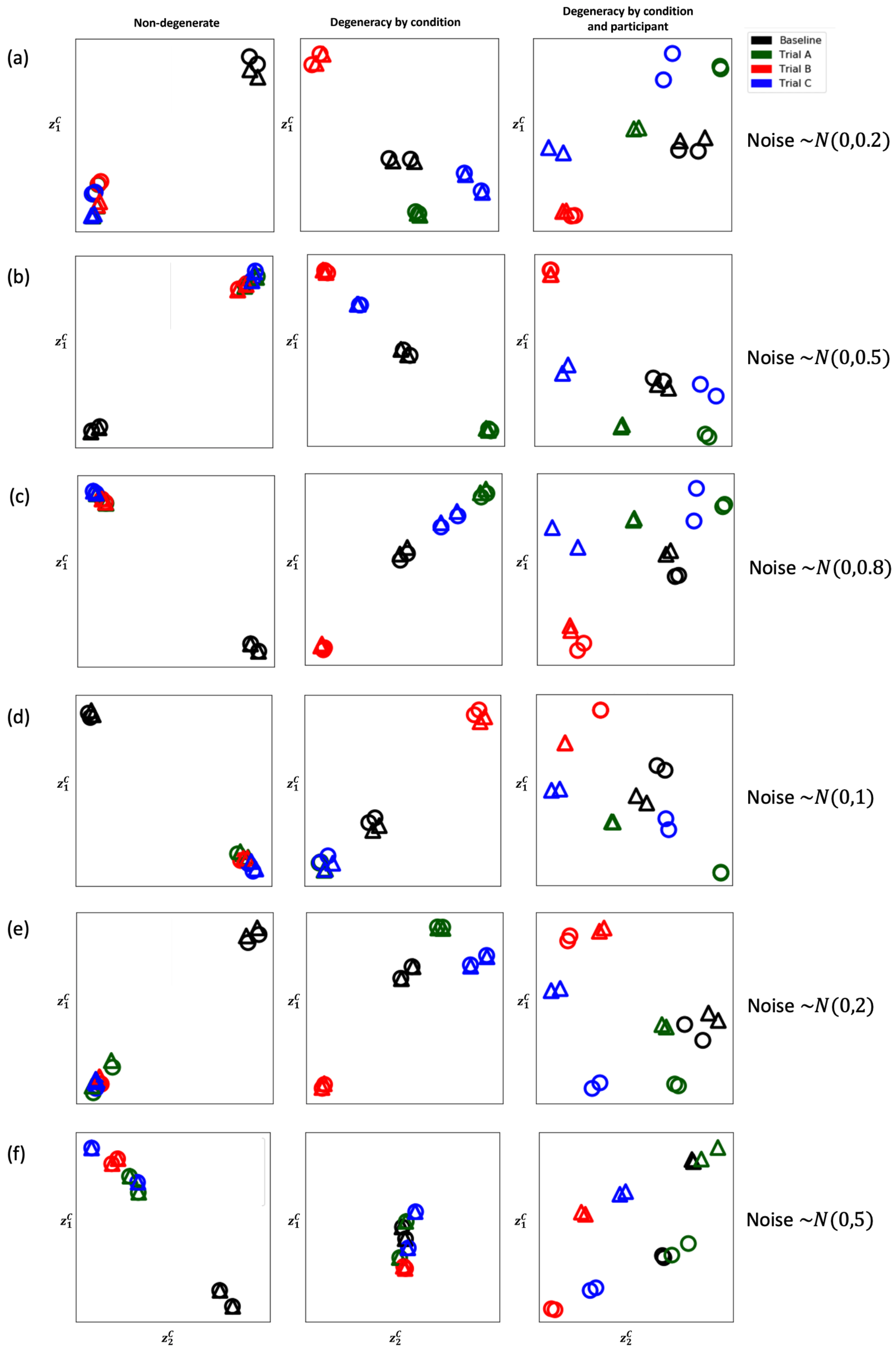

Figure B.3: Inferred embeddings using NTFA on data from three scenarios with additive Gaussian noise sampled from **(a)** $\mathcal{N}(0, 0.2)$, **(b)**$\mathcal{N}(0, 0.5)$, **(c)** $\mathcal{N}(0, 0.8)$, **(d)** $\mathcal{N}(0, 1)$, **(e)** $\mathcal{N}(0, 2)$, and **(f)** $\mathcal{N}(0, 5)$. The mean magnitude of the signal in our case was around 0.8.
